# Supplementary material for: Bacterial diversity dominates variable macrophage responses of tuberculosis patients in Tanzania
Source: Sci Rep. 2024 Apr 23;14:9287. doi: 10.1038/s41598-024-60001-0 (PMC11039734; doi:10.1038/s41598-024-60001-0)
Supplement: Supplementary file 1 — Supplementary Information. [file 41598_2024_60001_MOESM1_ESM.docx]

**Supplementary material**

**Supplementary table 1:** Patient characteristics categorized by lineages originally infecting the TB patients for specimen used for plasma analysis

| Characteristics | All participants  (n=92) | Lineage 1  (n=23) | Lineage 2  (n=23) | Lineage 3  (n=23) | Lineage 4  (n=23) |
| --- | --- | --- | --- | --- | --- |
| Age in years, median (IQR) | 31(25-39) | 37(26-45) | 30(24-35) | 29 (23-36) | 32(25-40) |
| Age group in years, n (%) |  |  |  |  |  |
| 18-24 | 23(25) | 3(13.0) | 7(30.4) | 7(30.4) | 6(26.1) |
| 25-34 | 33(36) | 7(30.4) | 9(39.1) | 9(39.1) | 8(34.8) |
| 35-44 | 24(26) | 6(26.1) | 6(26.1) | 6(26.1) | 6(26.1) |
| ≥45 | 12(13) | 7(30.4) | 1(4.3) | 1(4.3) | 3(13.0) |
| Male, n (%) | 71(77) | 18(78) | 19(83) | 20(87) | 14(61) |
| BMI, kg/m^2^, median (IQR) | 18.24(16.85-19.70) | 17.99(16.53-19.81) | 18.81(16.66-19.50) | 18.42(17.40-19.81) | 18.04(16.90-19.49) |
| Smoking | 26 (28) | 6(26) | 8(35) | 11(48) | 1(4) |
| HIV infected | 2(2.2) | 1(4) |  |  | 1(4) |
| On ART, n (%) of HIV infected | 2(100) | 1(100) |  |  | 1(100) |
| Symptoms ^1^, n (%) |  |  |  |  |  |
| Cough | 92(100) | 23(100) | 23(100) | 23(100) | 23(100) |
| Fever | 63(68) | 17(74) | 13(57) | 14(61) | 19(83) |
| Night sweat | 59(64) | 13(57) | 14(61) | 15(65) | 17(74) |
| Significant weight loss | 74(80) | 20(87) | 16(70) | 17(74) | 21(91) |
| TB patient category, n (%) |  |  |  |  |  |
| New | 87(95) | 22(96) | 20(87) | 23(100) | 22(96) |
| Relapse | 4(4) | 1(4) | 2(9) | na | 1(4) |
| Treatment after default | 1(1) | na | 1(4) | na | na |
| Full blood counts^1^ (10^9^ cells/L) |  |  |  |  |  |
| White Blood cells, median (IQR) | 7(5.76-8.79) | 7.02(5.51-9.13) | 7.39 (5.94- 9.18) | 7.38 (6.12-8.17) | 6.62(5.15-8.66) |
| Platelets, median (IQR) | 337 (269- 432) | 339 (247-429) | 411 (299-468) | 308 (272-402) | 303(254-389) |
| Red blood cells, mean (±SD) | 4.61 (0.91) | 4.43 (0.89) | 4.51 (1.06) | 4.65(0.84) | 4.85 (0.83) |
| Xpert, Ct value, median (IQR) | 18.3 (15.6-21.5) | 18.10 (15.55-20.65) | 19.60(16.60-23.35) | 18.30 (16.40 -21.45) | 18.20 (16.65-21.90) |
| Culture results, n (%) |  |  |  |  |  |
| Scanty | 10(11) | 3(13) | 1(4) | 2(9) | 4(17) |
| 1^+^ | 54(58) | 9(39) | 15(65) | 17(74) | 13(57) |
| 2^+^ | 22(24) | 9(39) | 5(22) | 4(17) | 4(17) |
| 3^+^ | 6(7) | 2(9) | 2(9) | na | 2(9) |

BMI-Body mass index, Culture results; Scanty: < 20 colonies, 1**^+^**: 20 to 200 colonies, 2**^+^**: > 200 discrete colonies, 3**^+^**: > 200 confluent colonies. na: not applicable

**Supplementary table 2:** Controls (Xpert MTB/RIF^®^ negative and culture negative) socio-demographic characteristics

| Characteristics | controls  (n=20) |
| --- | --- |
| Age in years, median (IQR) | 33 (29-40) |
| Age group in years, n (%) |  |
| 18-24 | 1(5) |
| 25-34 | 11(55) |
| 35-44 | 7(35) |
| ≥45 | 1(5) |
| Male, n (%) | 16(80) |
| BMI, kg/m^2^, median (IQR) | 20.32(18.10-22.62) |
| Smoking | 14 (70) |
| HIV status negative | 20(100) |
| Symptoms, n (%) |  |
| Cough | 6(30) |
| Fever | 1(5) |
| Night sweat | nil |
| Significant weight loss | 1(5) |


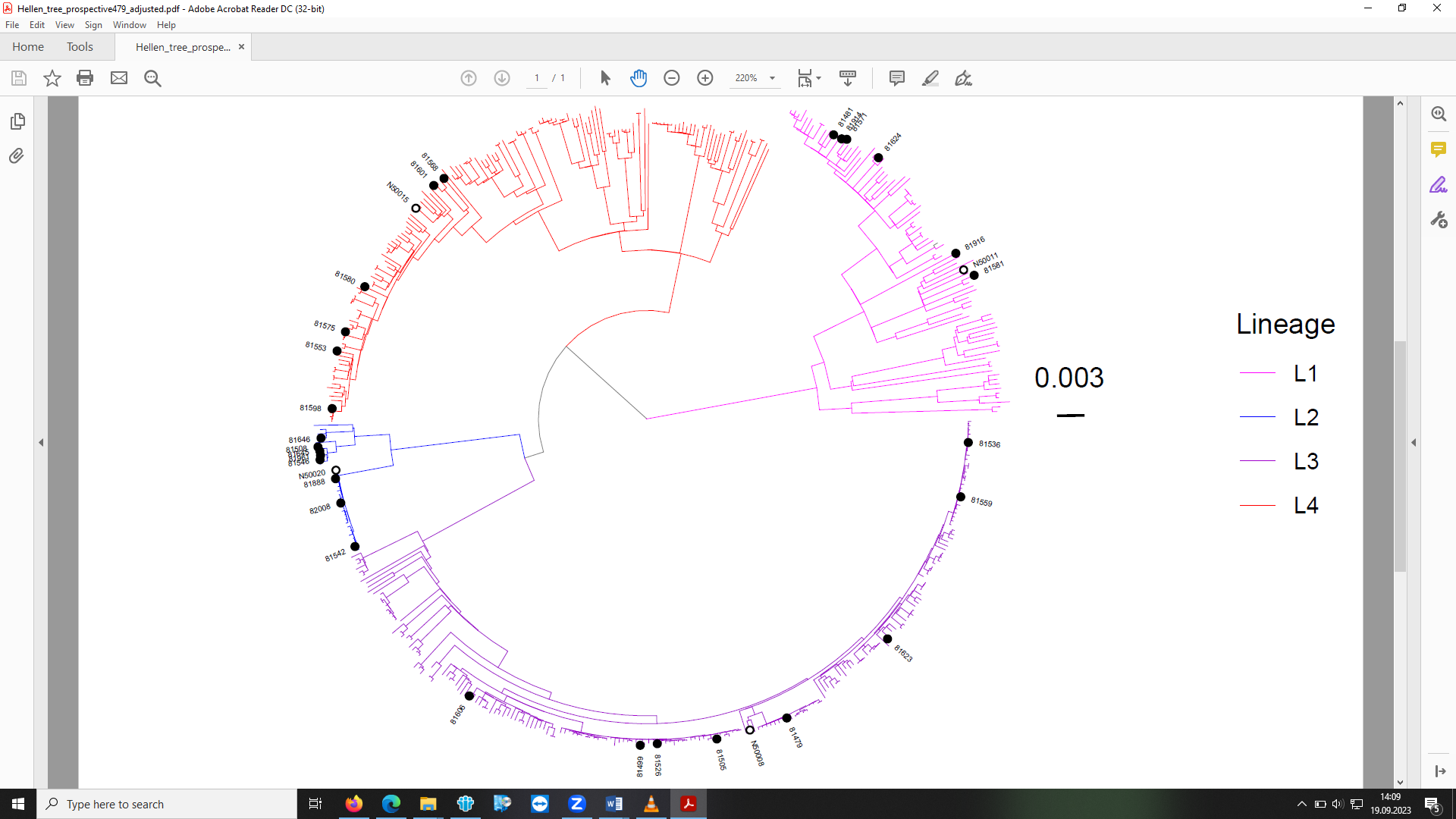


**Supplementary figure 1:** Phylogenetic tree of 479 isolates showing the genetic relationship between the strains used in our study highlighted with opened circles and labelled as N50008, N50011, N50015, and N50020. The tree is rooted with a M. canettii strain (SAMN00102920) as an outgroup and the scale bar represents the number of substitutions per site. Branches are colored according to the MTBC lineage, strains isolated from patients from which monocyte-derived macrophages were prepared are labeled with heavy black circles, and strains used for MDM infections are labelled with black circles. The phylogenetic tree was constructed with RAxML v 8.2.11 using the general time-reversible model of sequence evolution (options –m GTRCAT –V) with a M. canettii strain (SAMN00102920) as an outgroup from alignments of variable positions with a maximum of 10% of missing data. Visualization was done using the R package ggtree.


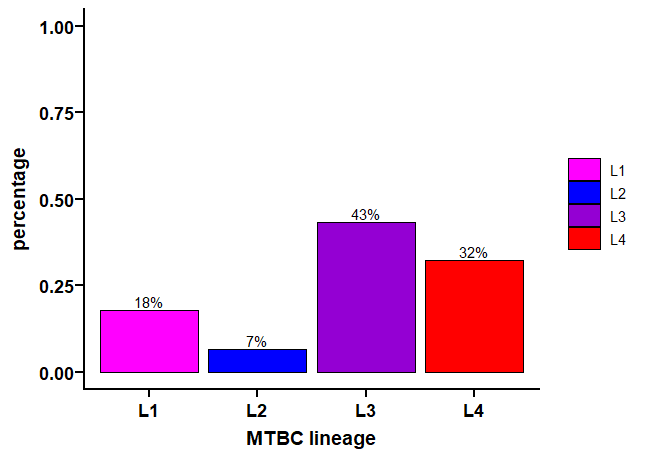


**Supplementary figure 2:** Distribution of MTBC lineages circulating in the Temeke district of Dar es Salaam in Tanzania. Lineage identification was done based on phylogenetic markers using WGS data of MTBC strains isolated from the sputa of 481 patients (ref Steiner A, Stucki D, Coscolla M, Borrell S, Gagneux S. KvarQ: targeted and direct variant calling from fastq reads of bacterial genomes. BMC Genomics. 2014;15:881. pmid:25297886).


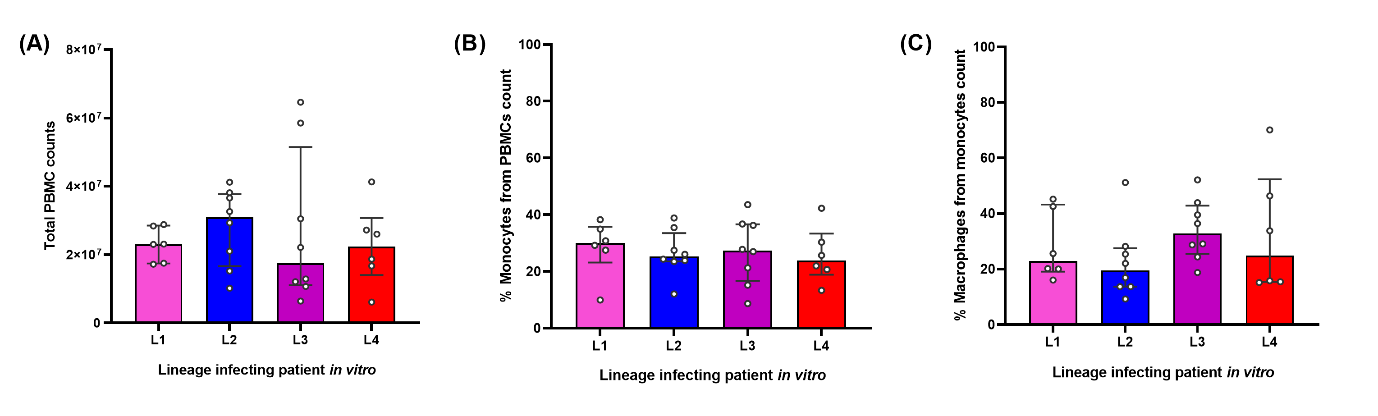


**Supplementary figure 3:** Summary of cell counts from patients infected with endemic strains in vitro. (a) Total PBMC counts. (b) Proportion of monocytes isolated from PBMCs using CD14 magnetic beads. (c) Proportion of macrophages differentiated from monocytes after 6 days using M-CSF.


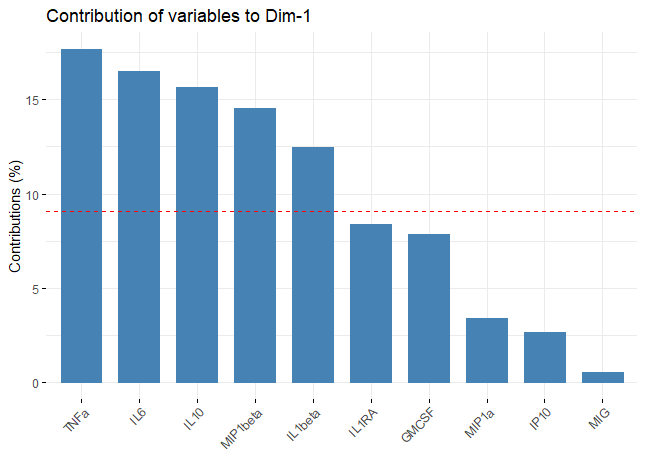


**Supplementary figure 4**: Bar plot of the contribution of each predictor variable to the first principal component.

**Supplementary figure 5:** CFUs of all inoculums were assessed for each infection experiment and did not differ statistically.


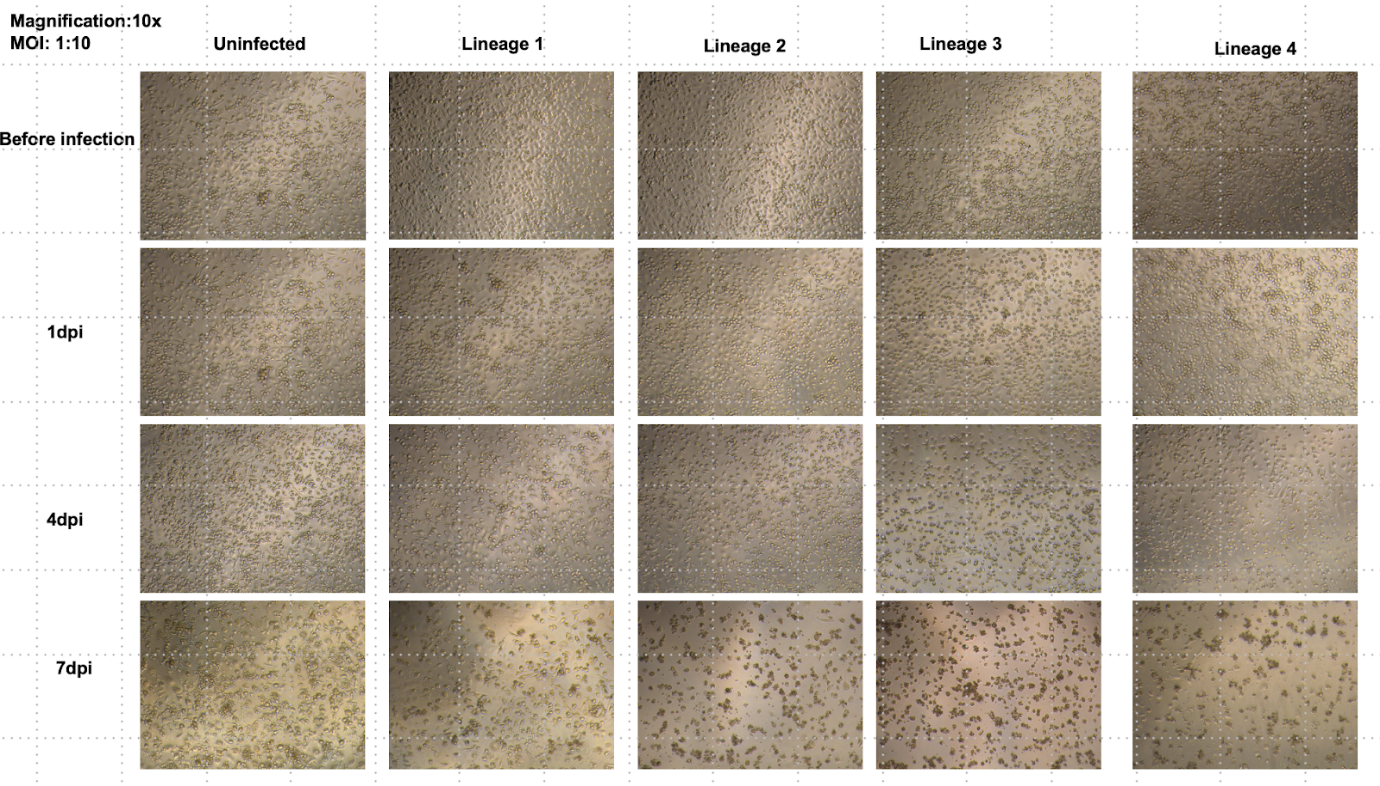


**Supplementary figure 6:** Representative overview of the cellular morphology monitored for each infection experiment at the indicated time point.

**Supplementary figure 7**: Symbols and lines dot plot of HMGB-1 concentration in the supernatants of patients’ MDMs after 24h of infection by the indicated representative lineage.
